# Elucidating Structure-Bioactivity Relationships of Methyl-Branched Alkanes in the Contact Sex Pheromone of the Parasitic Wasp *Lariophagus distinguendus*

**DOI:** 10.3390/insects4040743

**Published:** 2013-12-03

**Authors:** Stephan Kühbandner, Jan E. Bello, Kenji Mori, Jocelyn G. Millar, Joachim Ruther

**Affiliations:** 1 Institute of Zoology, University of Regensburg, Universitätsstraße 31, Regensburg D-93053, Germany; E-Mail: marsupials@web.de; 2 Department of Chemistry, University of California, Riverside, CA 92521, USA; E-Mails: jbell011@ucr.edu (J.E.B.); jocelyn.millar@ucr.edu (J.G.M.); 3 Photosensitive Materials Research Center, Toyo Gosei Co., Ltd., 4-2-1 Wakahagi, Inzai-shi, Chiba 270-1609, Japan; E-Mail: kjk-mori@arion.ocn.ne.jp; 4 Department of Entomology, University of California, Riverside, CA 92521, USA

**Keywords:** chemoreception, contact sex pheromone, cuticular hydrocarbons, *Lariophagus distinguendus*, 3-methylheptacosane, parasitic wasp, Pteromalidae

## Abstract

The exoskeletons of insects are covered by complex mixtures of cuticular hydrocarbons (CHCs) which are involved in social and sexual communication. However, little is known about the relationship between the structures of CHCs and their behavioral activity. The key component of the contact sex pheromone of the parasitoid *Lariophagus distinguendus* is 3-methylheptacosane (3-MeC27), which is present in CHC profiles of both females and newly emerged males. The CHCs of females and young males elicit wing-fanning behavior in older males. However, as young males age, 3-MeC27 disappears from their CHC profiles and they no longer elicit wing-fanning responses from other males. We applied enantiopure 3-MeC27 and structurally related CHCs (with respect to chain length or methyl-branch position) to the cuticle of aged male dummies and recorded the wing-fanning behavior of responding males. Only the two enantiomers of 3-MeC27 restored the dummies’ attractiveness. The addition of structurally related CHCs or various n-alkanes to bioactive dummies of young males and females significantly decreased wing-fanning by test males. Hence, *L. distinguendus* males respond specifically but not enantioselectively to 3-MeC27, and perceive the CHC profiles as a whole. Both removal (as is the case with 3-MeC27 in aging males) and addition of individual compounds may disrupt the behavioral response.

## 1. Introduction

Insects utilize chemical signals and cues in all aspects of their life histories and ecologies, and thus possess an innate ability to detect and discriminate different chemicals and associate them with the correct biological context. It has been well established that insects employ volatile substances for long-range communication, and more recently it has become clear that many insects also utilize non-volatile compounds as short-range or contact pheromones [1]. These compounds are components of the protective layer of cuticular lipids covering the insect exoskeleton. This lipid layer consists primarily of a complex blend of n-alkanes, methyl-branched alkanes, and alkenes, typically with chain lengths of about 21–37 carbons (referred to as cuticular hydrocarbons, CHCs), as well as more polar compounds such as long-chain fatty acids, alcohols, aldehydes, wax esters, and triacylglycerides [2,3,4,5]. CHCs function primarily as a water barrier preventing desiccation [6], but components of this protective layer are also utilized in insect communication [1]. Solitary insects use CHCs to recognize conspecifics and to determine gender, and thus identify potential mates [1,7,8]. CHCs are also employed as kairomones, fertility signals, and to mark territories [7,9]. In social insects, CHCs are directly involved in nestmate recognition, formation and maintenance of social castes, and determination of the health and fecundity of the reproductive caste [1].

The CHC profiles of insects can range from relatively simple mixtures of only a few compounds to complex blends of more than 100 substances [10,11]. However, little is known about how insects perceive and process the information that is encoded in the cuticular lipids [12,13,14,15]. It is assumed that insects use only a small subset of the cuticular compounds as semiochemicals [16,17,18,19,20] with the majority of CHC components being considered to have little or no communicative function [10,11,21]. The biological activity of methylalkanes and alkenes is directly correlated with their chain lengths and the positions of methyl branch points and double bonds, respectively [11,13,17,18,19,22]. This suggests that methylalkanes and alkenes are better suited for use as signal molecules than straight-chain alkanes because they possess additional structural features that provide for discrimination using criteria other than chain length alone [10,21,23]. Methylalkanes often occur on the insect cuticle as series of homologs, with methyl branch points at the same position in chains of variable length [11]. It is still unclear whether insects are able to discriminate such homologs or if they “generalize” them. In the latter case, methylalkanes differing only in chain length might convey the same amount of information and therefore be used as “synonyms” [11,13,19]. This would make CHC profiles functionally far less complex than one would expect from the mere number of compounds [11,24]. Further potential information might be encoded in the stereochemistry of methylalkanes [3,25,26] and the relative proportions in which they occur in the CHC profile of insects [10,12]. However, despite the substantial body of literature on the semiochemical functions of CHCs, many details on the relationships between structural features and bioactivity remain to be elucidated.

*Lariophagus distinguendus* Förster (Hymenoptera: Pteromalidae) is an idiobiont ectoparasitoid that parasitizes the larvae and pupae of several species of beetles that infest stored products [27,28]. Females produce a contact sex pheromone on their cuticles. Males are arrested by this pheromone and respond by performing stereotypical courtship behavior that includes high-frequency wing-fanning [29,30]. Interestingly, the pupae of both sexes as well as newly emerged males apparently produce the same pheromone blend as females, but young males deactivate the pheromone within 32 hours after emergence. This deactivation is accompanied by the loss of 3-methylheptacosane (3-MeC27) and some minor CHCs [31,32]. The mechanism behind the disappearance of 3-MeC27 from the aging male cuticle is not yet known, but it has been shown that males killed before the pheromone deactivation period retain the attractive hydrocarbon blend indefinitely [31]. Reapplication of synthetic 3-MeC27 onto the cuticle of aged males fully reinstates the pheromonal activity, so that they are courted by sexually mature males [3]. Thus, 3-MeC27 is a key component of the *L. distinguendus* contact sex pheromone. However, experiments using fractionated bioactive lipid extracts revealed that 3-MeC27 only elicits a response when it is presented in combination with a chemical background of the other CHCs and triacylglycerides that also occur on the cuticle of *L. distinguendus* wasps [3]. The results mentioned above have shown that the disappearance of a single compound from a bioactive CHC profile can terminate the wing fanning response of *L. distinguendus* males. It is not known, however, whether a bioactive CHC profile can also be disturbed by adding individual compounds as has been shown in the context of nestmate recognition in social insects [33].

In this study, we investigated the structure-bioactivity relationships of methyl-branched CHCs in *L. distinguendus*. In particular, we tested whether the responses of males to 3-MeC27 are specific with respect to chain length, position of the methyl branch, and absolute configuration. In addition, we tested the hypothesis that the responses elicited by bioactive CHC profiles, such as those of females and newly emerged males, can be disrupted by the addition of synthetic methylalkanes and n-alkanes to those cuticular profiles.

## 2. Materials and Methods

### 2.1. Insects

*Lariophagus distinguendus* wasps were reared on late instar larvae and prepupae of the granary weevil *Sitophilus granarius* (Curculionidae) at 25 °C and 40%–50% relative humidity under a photoperiod of 12 h:12 h light:dark [28]. Male wasps used as responders in bioassays were isolated shortly after emergence and kept separately for two days under the described rearing conditions. Two types of dead wasps were used as dummies to study the effects of added synthetic alkanes on the pheromonal activity of the wasps’ CHC profiles. The first type of dummies were males that had been isolated for four days and were subsequently freeze-killed (referred to as 4-d-old males). These males no longer elicit pheromonally induced wing-fanning responses from courting males [31] and were used in Experiment 1 (see below). The second type of dummies were males and females which were freeze-killed immediately after emergence (referred to as 0-d-old males/females). These dummies elicit intense wing-fanning behavior in responding males [31] and were utilized in Experiment 2. All dummies were stored at −23 °C, and were defrosted immediately prior to bioassays.

### 2.2. Synthesis of Reference Chemicals

#### 2.2.1. General Methods and Information for Synthesis

All solvents were Optima grade (Fisher Scientific, Pittsburgh, PA, USA). Tetrahydrofuran (THF) was distilled from sodium/benzophenone under argon. ^1^H- and ^13^C-NMR spectra were recorded with a Varian INOVA-400 (400 and 100.5 MHz, respectively) spectrometer (Palo Alto, CA, USA), as CDCl_3_ solutions. ^1^H-NMR chemical shifts are expressed in ppm relative to residual CHCl_3_ (7.27 ppm) and ^13^C-NMR chemical shifts are reported relative to CDCl_3_ (77.16 ppm). Unless otherwise stated, solvent extracts of reaction mixtures were dried over anhydrous Na_2_SO_4_ and concentrated by rotary evaporation under reduced pressure. Crude products were purified by vacuum flash chromatography or column flash chromatography on silica gel (230–400 mesh; Fisher Scientific). Yields refer to isolated yields of chromatographically pure products. Mass spectra were obtained with a Hewlett-Packard (HP) 6890 GC (Hewlett-Packard, Avondale, PA, USA) interfaced to an HP 5973 mass selective detector, in EI mode (70 eV) with helium as carrier gas. The GC was equipped with a DB17-MS column (25 m × 0.20 mm i.d., 0.33 µm film). Reactions with air- or water-sensitive reagents were carried out in oven-dried glassware under argon. 

#### 2.2.2. Synthesis of (*S*)-3-Methylnonacosane [(*S*)-**7**], (*S*)-3-Methylhentriacontane [(*S*)-**8**], (*R*)-3-Methylnonacosane [(*R*)-**7**], and (*R*)-3-Methylhentriacontane [(*R*)-**8**] (Figure 1)

(*S*)-2-Methyl-1-butanol **1** (1.21 g, 13.7 mmol, Alfa Aesar, Ward Hill, MA, USA) was dissolved in 15 mL of CH_2_Cl_2_ and cooled to −10 °C, then pyridine (1.1 mL, 13.7 mmol) and triflic anhydride (2.81 mL, 16.4 mmol) were added sequentially, and the resulting mixture was stirred for 1.5 h at −10 °C. The reaction was then diluted with 45 mL of pentane, filtered through a plug of silica gel, and concentrated to afford (*S*)-2-methylbutyl triflate **2** as a colorless oil in quantitative yield, which was used immediately in the next step without further purification [34].

(11-(*tert-*Butyldimethylsilyloxy)undecyl)magnesium bromide (1.5 M, 8.2 mL, 12.3 mmol) was prepared by dropwise addition of 11-(*tert-*butyldimethylsilyloxy)undecyl bromide (5.5 g, 15.02 mmol) to Mg turnings (365 mg, 15 mmol) in 10 mL of dry Et_2_O, followed by stirring 2 h at 23 °C. Triflate **2** (2.8 g, 12.7 mmol) was taken up in 40 mL of dry Et_2_O and cooled to −30 °C under argon, Li_2_CuCl_4_ (0.48 M, 1.45 mL, 0.69 mmol) was added dropwise, and the solution was stirred for 15 min. The freshly prepared Grignard reagent was then added dropwise over 30 min and the resulting mixture was stirred at −40 °C for 4 h. The reaction was then quenched with saturated aqueous NH_4_Cl (50 mL) and extracted with hexane. The hexane extract was washed with water and brine, dried and concentrated, and the residue was purified by column chromatography (Et_2_O/hexane, 1:19) to afford silyl ether **3** (3.35 g, 78%) as a colorless oil [35].

A solution of Ph_3_PBr_2_ (9.62 g, 22.8 mmol) was prepared by treatment of Ph_3_P (5.96 g, 22.8 mmol) with Br_2_ (1.17 mL, 22.8 mmol) in 100 mL CH_2_Cl_2_ at 0 °C. After warming to room temperature, silyl ether **3** (3.25 g, 9.1 mmol) in 20 mL dry CH_2_Cl_2_ was added slowly, the mixture was stirred for 1 h, then diluted with hexane (200 mL) and filtered through a plug of silica gel. After concentration, the crude product was purified by column chromatography (Et_2_O/Hex, 1:99) to afford alkyl bromide **4** (2.55 g, 92%) [36].

**Figure 1:**
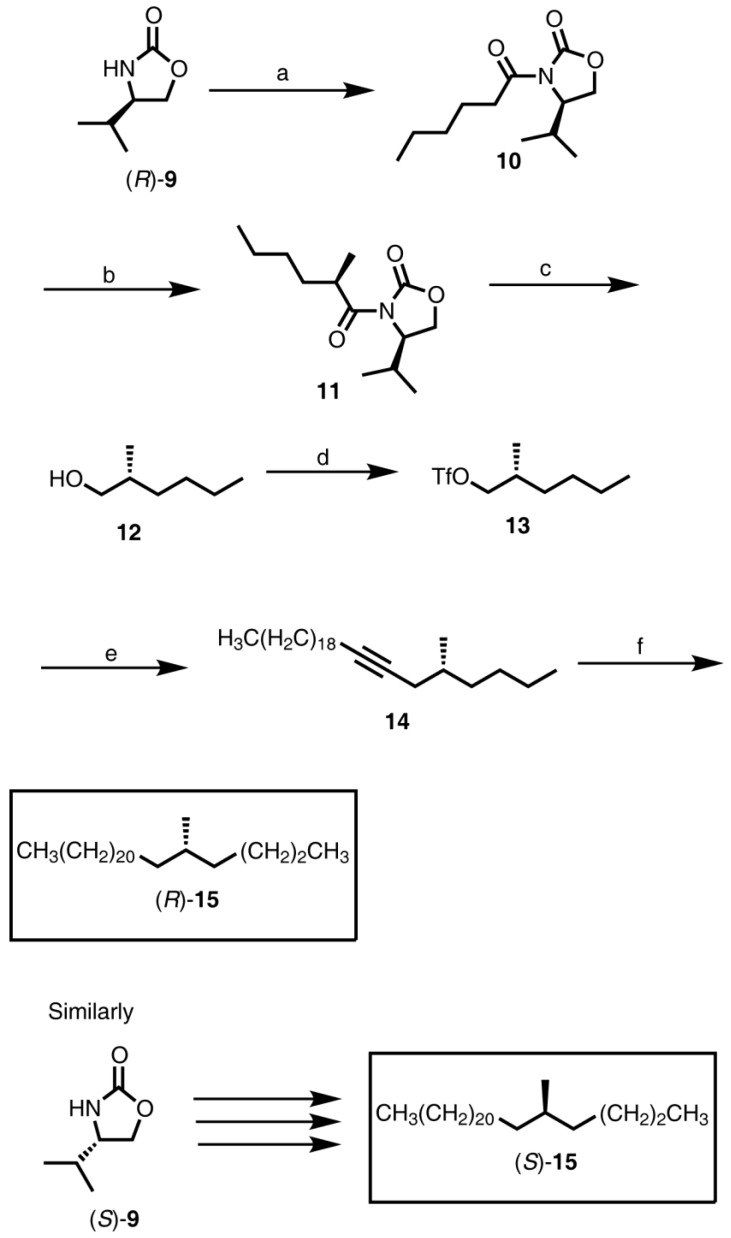
Synthesis of (*S*)-3-methylnonacosane [(*S*)-**7**], (*S*)-3-methylhentriacontane [(*S*)-**8**], (*R*)-3-methylnonacosane [(*R*)-**7**], and (*R*)-3-methylhentriacontane [(*R*)-**8**]. Reagents: (**a**) Tf_2_O, pyridine, CH_2_Cl_2_ (quantitative); (**b**) (11-(*tert*-butyldimethylsilyloxy)undecyl)-magnesium bromide, Li_2_CuCl_4_, Et_2_O (78%); (**c**) Ph_3_PBr_2_, CH_2_Cl_2_ (91.5%); (**d**) tetradecynyl lithium for **7**, hexadecynyl lithium for **8**, THF (82% and 85%, respectively); (**e**) 5% Rh/C, H_2_, hexane (98%).

1-Tetradecyne (416 mg, 2.14 mmol, Farchan/GFS Chemicals, Powell, OH, USA) was dissolved in 15 mL dry THF in a 3-necked flask under Ar and after cooling to −78 °C, *n*-BuLi (2.2 M in hexanes, 972 µL, 2.14 mmol) was added dropwise over 10 min. The reaction was stirred for 30 min at −78 °C, then warmed to 23 °C. Alkyl bromide **4** (600 mg, 2.04 mmol) and NaI (50 mg, 0.24 mmol) were then added and the mixture was refluxed for 8 h. The mixture was then cooled to 23 °C, quenched with saturated aqueous NH_4_Cl, and extracted with hexane. The hexane extract was washed with water and brine, dried, and concentrated. Unreacted 1-tetradecyne was removed by Kugelrohr distillation of the crude product (oven temp. 50 °C, 0.1 mm Hg), affording (*S*)-27-methylnonacos-13-yne (*S*)-**5** (746 mg, 82%) as a colorless oil [37]. The oil was added to a slurry of 5% Rh/C (80 mg) and anhydrous Na_2_CO_3_ (700 mg, 5.2 mmol) in hexane (10 mL) and stirred for 10 h under a slight positive pressure of H_2_. The mixture was filtered through a plug of silica gel and concentrated to afford 763 mg of crude crystalline (*S*)-3-methylnonacosane [38].

Recrystallization from hexane/acetone (1:5) gave 737 mg of pure (>98%) (*S*)-**7** in 53% overall yield in 5 steps, mp 34 °C, [α]_D_^23^ = +3.63° (c = 1.52, CH_2_Cl_2_). ^1^H-NMR (CDCl_3_), ∂_H_ (ppm): 0.84 (3H, d, *J* = 6.3 Hz), 0.85 (3H, t, *J* = 6.7 Hz), 0.87 (3H, t, *J* = 6.5 Hz), 1.16–1.4 (53 H, broad m). ^13^C-NMR, ∂_c_ (ppm): 11.62, 14.32, 19.45, 22.91, 25.67, 27.36, 29.58, 29.72, 29.93, 30.25, 31.81, 32.16, 34.62, 36.88. MS (EI, 70 eV, *m*/*z*, relative abundance): 422 (1, M^+^), 407 (1), 393 (12), 379 (1), 365 (1), 351 (1), 337 (1), 323 (1), 309 (2), 295 (2), 281 (2), 267 (2), 253 (2), 239 (3), 225 (3), 211 (2), 197 (3), 183 (3), 169 (5), 155 (5), 141 (7), 127 (10), 113 (11), 99 (19), 85 (30), 71 (43), 57 (99), 43 (100).

(*S*)-3-Methylhentriacontane [(*S*)-**8**] was prepared in analogous fashion in 51% yield by substitution of hexadecynyllithium for tetradecynyllithium in the 4th reaction. 1-Hexadecyne was obtained from treatment of 1-tetradecyl bromide with lithium acetylide-ethylene diamine complex in DMSO [39]. Mp = 36 °C, [α]_D_^23^ = +3.43° (c = 1.55, CH_2_Cl_2_). ^1^H-NMR (CDCl_3_) ∂_H_ (ppm): 0.84 (3H, d, *J* = 6.5 Hz), 0.85 (3H, t, *J* = 6.7 Hz), 0.87 (3H, t, *J* = 6.8 Hz), 1.16–1.4 (57 H, broad m). ^13^C-NMR, ∂_c_ (ppm): 11.59, 14.32, 19.41, 22.87, 25.48, 27.36, 29.28, 29.58, 29.73, 29.94, 30.25, 31.81, 32.16, 34.62, 36.88. MS (EI, 70 eV, *m*/*z*, relative abundance): 450 (1, M^+^), 435 (2), 421 (43), 407 (1), 393 (17), 379 (1), 365 (1), 351 (1), 337 (1), 323 (1), 309 (1), 295 (2), 281 (2), 265 (3), 253 (3), 239 (3), 225 (3), 211 (5), 197 (5), 183 (5), 169 (5), 155 (7), 141 (9), 127 (13), 133 (17), 99 (30), 85 (39), 71 (52), 57 (99), 43 (100).

(*R*)-3-methylnonacosane [(*R*)-**7**] (52% overall yield, purity >97%) was prepared in analogous fashion to (*S*)-**7** by substitution of (*R*)-2-methyl-1-butanol for (*S*)-2-methyl-1-butanol in the 1st reaction. (*R*)-2-Methyl-1-butanol was obtained by enzymatic resolution of racemic 2-methyl-1-butanol with Amano *Pseudomonas fluorescens* lipase (Aldrich Chemical Co., Milwaukee, WI, USA) and vinyl acetate in dry CH_2_Cl_2_ [40]. Mp = 33 °C, [α]_D_^23^ = −3.53° (c = 1.52, CH_2_Cl_2_). Its spectroscopic data were equivalent to those of (*S*)-**7**.

(*R*)-3-methylhentriacontane [(*R*)-**8**] (50% overall yield, purity >99%) was prepared in analogous fashion to (*S*)-**8** by substitution of (*R*)-2-methyl-1-butanol for (*S*)-2-methyl-1-butanol in the 1st reaction. Mp = 36 °C, [α]_D_^23^ = −3.37° (c = 1.53, CH_2_Cl_2_). Its spectroscopic data were equivalent to those of (*S*)-**8**.

#### 2.2.3. Synthesis of (*R*)-5-Methylheptacosane [(*R*)-**15**] and (*S*)-5-Methylheptacosane [(*S*)-**15**] (Figure 2)

(*R*)-4-Isopropyloxazolidin-2-one **9** (2.25 g, 17.7 mmol) [41] was dissolved in dry THF (70 mL) and cooled to −78 °C, *n*-BuLi (2.89 M in hexanes, 6.4 mL, 18.5 mmol) was added dropwise over 10 min and the reaction was stirred for 1 h. Hexanoyl chloride (2.82 mL, 19.5 mmol) was then added dropwise and the resulting mixture was stirred at −78 °C for 20 min, then warmed to 0 °C for 1.5 h. The reaction was quenched with 1 M aqueous K_2_CO_3_ (50 mL) and extracted with hexane. The hexane extract was washed with water and brine, dried and concentrated, and the residue was purified by column chromatography (EtOAc/hexanes, 1:9) to afford oxazolidinone amide **10** (3.91 g, 96%) as a colorless oil [42].

A solution of **10** (3.0 g, 12.95 mmol) in dry THF was cooled to −78 °C under Ar and sodium hexamethyldisilazide (NaHMDS, 2.0 M in THF, 7.12 mL, 14.25 mmol) was added dropwise over 15 min. The reaction was stirred at −78 °C for 1 h, then MeI (3.22 mL, 52 mmol) was added dropwise, and the resulting solution was stirred at −78 °C for 2 h. The reaction was quenched with saturated aqueous NH_4_Cl (75 mL) and extracted with hexane. The hexane extract was washed sequentially with 1 M HCl, saturated NaHCO_3_, and brine, then dried and concentrated. The residue was purified by column chromatography to afford ((*R*)-2-methylhexanoyl)oxazolidinone (**11**) (3.01 g, 94%) as a colorless oil [43].

**Figure 2:**
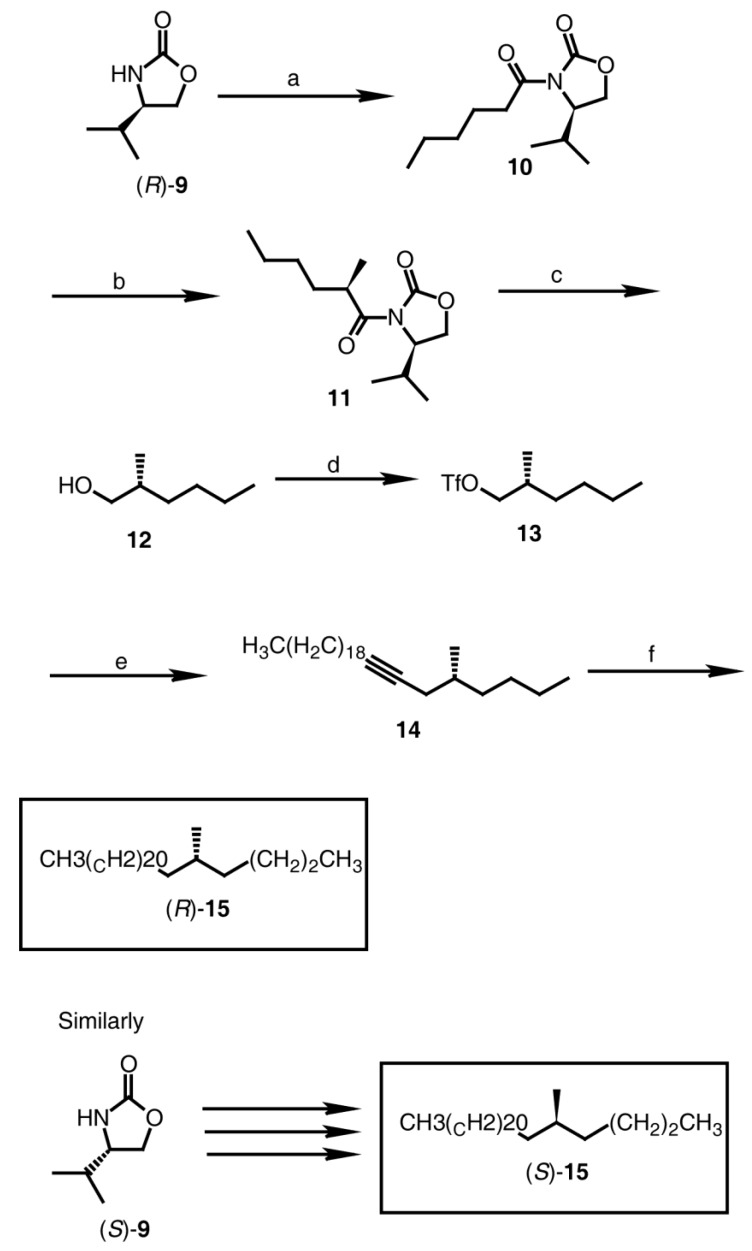
Synthesis of (*R*)- and (*S*)-5-methylheptacosane (**15**). Reagents: (**a**) i. *n-*BuLi, THF; ii. CH_3_(CH_2_)_4_COCl (96%); (**b**) i. NaHMDS, THF; ii. MeI (94%); (**c**) LiBH_4_, Et_2_O (92%); (**d**) Tf_2_O, pyridine, CH_2_Cl_2_ (quantitative for **7**); (**e**) heneicosynyl lithium, THF (76%–80%); (**f**) H_2_ (1 atm), 5% Rh/C, hexane (96%–97%).

A solution of ((*R*)-2-methylhexanoyl)oxazolidinone (**11**) (3.0 g, 12.9 mmol) in Et_2_O (80 mL) was cooled to 0 °C, and dry MeOH (1.9 mL, 25.3 mmol) was added, followed immediately by LiBH_4_ (562 mg, 25.8 mmol). The reaction was stirred at 0 °C for 3 h, then quenched with saturated NaHCO_3_ (60 mL), and extracted with Et_2_O. The ether extract was washed with saturated NH_4_Cl, water and brine, and after drying and concentration, the residue was purified by column chromatography (EtOAc/hexane 1:5) to afford alcohol **12** (1.41 g, 92%) as a colorless oil [44].

Alcohol **12** (500 mg, 4.3 mmol) was treated sequentially with pyridine (346 µL, 4.3 mmol) and triflic anhydride (880 µL, 5.16 mmol) in CH_2_Cl_2_ (20 mL) at −10 °C for 2 h. The reaction then was diluted with pentane (60 mL) and filtered through a plug of silica gel, rinsing with 3:1 hexane:CH_2_Cl_2_. Concentration of the filtrate gave alkyl triflate **13** (1.07 g, quantitative) as a colorless oil, which was used immediately without further purification [34].

A solution of 1-heneicosyne (1.23 g, 4.2 mmol) [45] in 10 mL of dry THF was cooled to −10 °C, *n*-BuLi (2.89 M in hexanes, 1.46 mL, 4.22 mmol) was added dropwise over 10 min, and the reaction was stirred for 1 h. Alkyl triflate **13** (1.07 g, 4.3 mmol) in 5 mL THF was then added by syringe pump over 30 min, and the reaction was stirred at −10 °C for 5 h. The reaction was quenched with water (20 mL) and extracted with hexane. The hexane extract was washed with brine, dried and concentrated, and the residue was purified by vacuum flash chromatography (hexane) to afford (*R*)-5-methylheptacos-7-yne [(*R*)-**14**] (1.35 g, 80%) [46].

(*R*)-5-methylheptacos-7-yne [(*R*)-**14**] (1.35 g, 3.44 mmol) was added to a slurry of 5% Rh/C (135 mg) and anhydrous Na_2_CO_3_ (1.09 g, 10.3 mmol) in hexane (15 mL) [38]. The reaction was stirred under a slight positive pressure of H_2_ for 8 h, then filtered through a plug of silica gel to afford crude (*R*)-5-methylheptacosane. After concentration, the residue was dissolved in boiling acetone (10 mL) and the solution was cooled to −20 °C. Filtration and vacuum drying yielded pure (*R*)-5-methylheptacosane [(*R*)-**15**] (1.29 g, 96%) as white waxy crystals in 64% overall yield in 6 steps. Mp = 32 °C, [α]_D_^23^ = −0.77° (c = 1.33, CHCl_3_). ^1^H-NMR (CDCl_3_), ∂_H_ (ppm): 0.83 (3H, d, *J* = 6.3 Hz), 0.85 (3H, t, *J* = 6.7 Hz), 0.87 (3H, t, *J* = 6.5 Hz), 1.16–1.4 (49 H, broad m). ^13^C-NMR, ∂_c_ (ppm): 11.52, 14.14, 19.72, 22.61, 23.67, 27.09, 29.38, 29.70, 29.93, 30.35, 31.81, 32.16, 34.62, 36.73. MS (EI, 70 eV, *m*/*z*, relative abundance): 394 (M^+^, 1), 365 (3), 337 (39), 308 (12), 295 (1), 281 (1), 253 (3), 225 (3), 197 (1), 183 (2), 169 (2), 155 (1), 141 (9), 112 (35), 85 (42), 71 (98), 57 (100), 43 (50).

(*S*)-5-methylheptacosane [(*S*)-**15**] (61% yield, purity >99%) was prepared in analogous fashion by substitution of (*S*)-4-isopropyloxazolidin-2-one [(*S*)-**9**] [36] for (*R*)-4-isopropyloxazolidin-2-one [(*R*)-**9**] in the first reaction, mp = 31.5 °C, [α]_D_^23^ = +0.73° (c=1.35, CH_2_Cl_2_). Its spectroscopic data were analogous to those of (*R*)-**15**.

#### 2.2.4. Synthesis of (*R*)- and (*S*)-7-Methylheptacosane

The enantiomers of 7-methylheptacosane were synthesized as previously described [47].

#### 2.2.5. Synthesis of (*R*)-and (*S*)-3-Methylpentacosane, and (*R*)- and (*S*)-3-Methylheptacosane

The enantiomers of 3-methylpentacosane and 3-methylheptacosane were synthesized as previously described [48].

### 2.3. Bioassays

#### 2.3.1. General Procedures for Bioassays

Bioassays were performed in a round test arena (diameter: 10 mm; height: 3 mm) as described previously [29]. Aliquots of 1 µL containing 150 ng of synthetic compounds (treatment) or the pure solvent (dichloromethane, control) were applied evenly to the cuticle of individual dummies with a 5 µL syringe (Hamilton, Bonaduz, Switzerland). After the solvent had evaporated for 2 min, treated dummies were transferred to the test arena and the total duration of wing-fanning of a test male was recorded during the following 5 min using a stereo microscope and The Observer XT 9.0 scientific software (Noldus Information Technology, Wageningen, The Netherlands). Each male was tested twice, first with a control dummy and subsequently with a treated dummy. Test males that did not perform wing-fanning behavior towards the dummy in a given bioassay were additionally exposed to a 0-d-old female dummy as a positive control to make sure that they were responsive. Data from those few males (<1% of all tested males) that did not respond to this positive control were discarded. All experiments were conducted with a sample size of 20 replicates (N = 20). After every replicate, the test arena was thoroughly cleaned with ethanol.

#### 2.3.2. Experiment 1: Structure-Bioactivity Relationship of Methylalkanes for the Restoration of the Pheromone in 4-d-Old Male Dummies

This experiment was performed to determine if other structurally related methylalkanes, differing in chain length or methyl-branch position, could mimic the pheromonal activity of 3-MeC27 when added to the cuticle of 4-d-old male dummies. For this purpose, the following enantiomerically pure methylalkanes (synthesized as described above) were applied at doses of 150 ng each to the cuticle of 4-d-old male dummies: (*R*)- and (*S*)-enantiomers respectively of 3-MeC25, 3-MeC29, 3-MeC31 (correct position of the methyl branch, differing chain length), and 5-MeC27 and 7-MeC27 (correct chain length, differing position of the methyl branch). (*R*)- and (*S*)-3-MeC27 also were tested as positive controls. The dose of 150 ng for all compounds was chosen because it is the approximate amount of 3-MeC27 found on the cuticle of female wasps [31]. All compounds tested in this experiment are minor components of the *L. distinguendus* CHC profile [31]. The absolute configurations of the natural products are unknown.

#### 2.3.3. Experiment 2: Interruption of Pheromone Activity in 0-d-Old Male and Female Dummies by the Addition of Individual CHCs

The disappearance of 3-MeC27 from the cuticle of aging males results in the deactivation of the contact pheromone response. Therefore we tested whether specific changes to the bioactive CHC profiles of newly emerged male and female dummies, such as the addition of isomers and homologs of 3-MeC27, could inhibit or interrupt the responses of courting males. For this purpose, the following methylalkanes were applied individually at doses of 150 ng to 0-d-old male or female dummies: (*R*)- and (*S*)-enantiomers of 3-MeC29, 3-MeC31, 5-MeC27, and 7-MeC27. Additionally, we tested whether the addition of straight chain alkanes (150 ng n-C27, n-C29, or n-C31) or an excess of the key component 3-MeC27 (150 ng of the (*R*)- or (*S*)-enantiomer) added to the cuticle of otherwise attractive 0-d-old male dummies, affected the wing-fanning behavior of test males. All n-alkanes tested in this experiment are minor components of the *L. distinguendus* CHC profile [31].

#### 2.3.4. Statistical Analysis

Data did not meet the assumptions for parametric statistical analysis. Therefore, non-parametric Wilcoxon signed rank tests were used for the comparison of the duration of wing-fanning exhibited by responding males towards different treatments (addition of a given synthetic alkane) and the corresponding solvent controls. For statistical calculations, the software R version 2.15.1 [49] was used.

## 3. Results

### 3.1. Experiment 1: Structure-Bioactivity Relationship of Methylalkanes for the Restoration of the Pheromone in 4-d-Old Male Dummies

**Figure 3:**
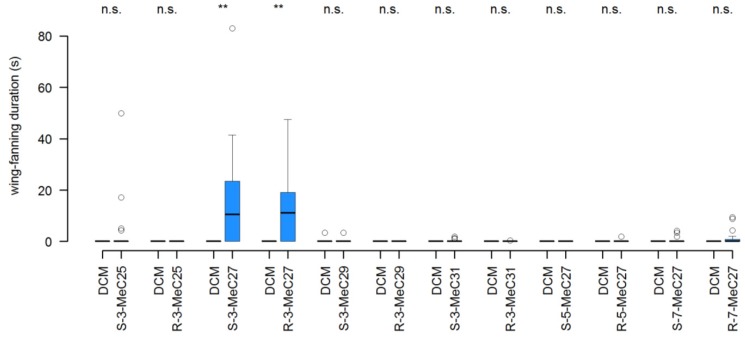
Restoration of pheromone activity in 4-d-old male dummies (Experiment 1). Wing-fanning duration during a 5-min observation period performed by *L. distinguendus* responder males towards 4-d-old male dummies treated with dichloromethane (DCM, control = white) and with 150 ng of different methyl-branched alkanes in dichloromethane, respectively (blue). Box-and-whisker plots show median (horizontal line), 25–75 percent quartiles (box), maximum/minimum range (whiskers) and outliers (>1.5× above box height). Asterisks indicate significant differences between a methylalkane treatment and the corresponding DCM control (*p* > 0.05 = non-significant (n.s.), *p* < 0.01 = **, Wilcoxon signed rank test; N = 20).

The addition of either (*R*)- or (*S*)-3-MeC27 to unattractive 4-d-old male dummies restored the wing-fanning behavioral responses elicited from test males, with responding males wing-fanning for significantly longer periods in the presence of pheromone-treated dummies than in the presence of solvent-treated controls (Figure 3). None of the other compounds when applied at doses of 150 ng to 4-d-old male dummies affected the wing-fanning behavior of test males (Figure 3). Thus, males specifically detected and responded to the key component 3-MeC27, but did not distinguish between the enantiomers when they were applied to 4-d-old dummies.

### 3.2. Experiment 2: Interruption of Pheromone Activity in 0-d-Old Male and Female Dummies by the Addition of Individual CHCs

**Figure 4:**
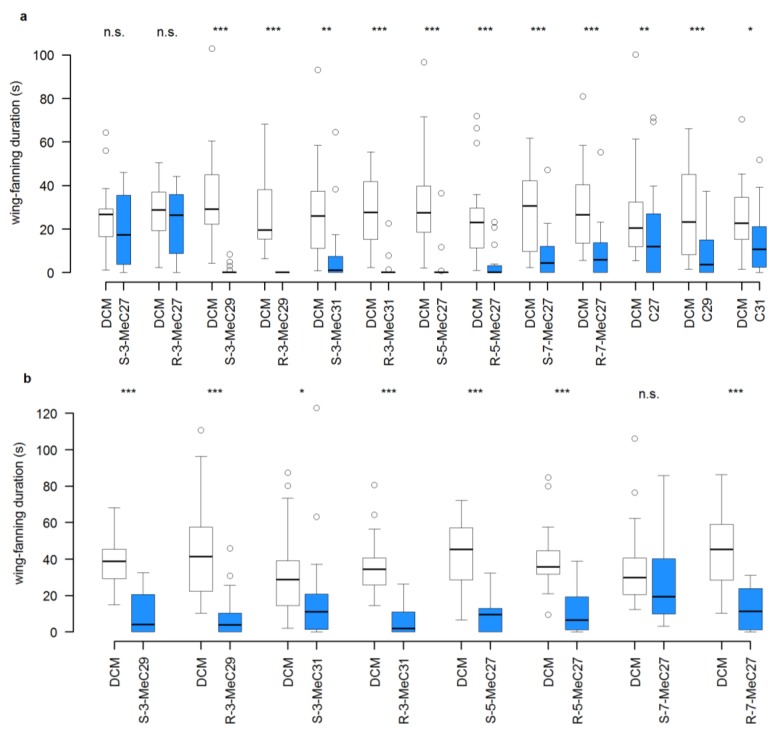
Interruption of pheromone bioactivity in 0-d-old male and female dummies (Experiment 2). Wing-fanning duration during a 5-min observation period performed by *L. distinguendus* responder males towards 0-d-old (**a**) male and (**b**) female dummies treated either with dichloromethane (DCM, control = white) or 150 ng of methyl-branched and straight-chain alkanes, respectively (blue). Box-and-whisker plots show median (horizontal line), 25–75 percent quartiles (box), maximum/minimum range (whiskers) and outliers (>1.5× above or below box height). Asterisks indicate significant differences (*p* > 0.05 = non-significant (n.s.), *p* < 0.05 = *, *p* < 0.01 = **, *p* < 0.001 = ***) between alkane treatment and the corresponding DCM control (Wilcoxon signed rank test; N = 20).

The application of 150 ng of any of the tested straight chain or methyl-branched alkanes other than (*R*)- or (*S*)-3-MeC27 onto bioactive, wing-fanning inducing 0-d-old male dummies resulted in a significant decrease in the duration of wing-fanning in test males (Figure 4a). Similar results were found for the methylalkanes when added to 0-d-old female dummies, except for (*S*)-7-MeC27, for which the decrease in wing-fanning duration was not statistically significant when compared to the solvent control (Figure 4b).

## 4. Discussion

The results of the present study in combination with previous work on *L. distinguendus* [3,31,50] shed new light on the role of CHCs as contact sex pheromones by showing that the CHC profile is perceived as a whole by males of this species. This is in contrast to the CHC-based contact sex pheromones of some other insects, in which individual methylalkanes elicit behavioral responses [18,19,20,51,52,53,54]. In *L. distinguendus*, both the removal as well as the addition of individual components to bioactive CHC profiles disrupted the behavioral response of the receiver. Under natural conditions, conspecific males stop responding to aging males as the major contact sex pheromone component, 3-MeC27, disappears from their cuticle. The evolution of this process has presumably been driven by the fitness costs imposed on young males by the courtship activities of conspecific males [50] and has been the prerequisite for the sex-specific conveyance of information. The deactivation of the pheromone in older males was shown to be reversible experimentally by the addition of synthetic 3-MeC27. Furthermore, the response was very specific to 3-MeC27 because when equal amounts of structurally related methylalkanes with differing chain lengths or methyl branch positions were applied to dummies, they did not restore the wing-fanning response. Thus, *L. distinguendus* males respond very specifically to 3-MeC27 and can discriminate variations in chain length of two carbons, and variations in methyl branch position of two or more positions. These results suggest that a missing key component in the CHC profile cannot be replaced by a structurally related analogue, and emphasizes the critical role of 3-MeC27 in the *L. distinguendus* contact sex pheromone. In contrast, the leaf beetle *Gastrophsa atrocyanea* has been shown to tolerate slight variations in the chain lengths and methyl branch points of methylalkanes in its contact pheromone without loss of bioactivity [18]. Similarly, in the longhorned beetle *Neoclytus acuminatus acuminatus*, three methylalkanes (7-MeC25, 7-MeC27 and 9-MeC27) differing in chain length or position of the methyl branch have been identified as the female’s contact sex pheromone. Each compound was active alone, but a combination of all three was required to elicit the full behavioral response from males.

The designation of 3-MeC27 as a key component of the contact sex pheromone of *L. distinguendus* is corroborated by the fact that treatment of attractive 0-d-old male dummies with an unnaturally high dose of synthetic 3-MeC27 resulted in no significant change in the wing-fanning responses elicited from courting males. In contrast, the application of any of the other synthetic methylalkanes onto 0-d-old male dummies resulted in a significant decrease in the wing-fanning response. The same was true when 0-d-old female dummies were treated with synthetic methylalkanes other than 3-MeC27, with the exception of those treated with (*S*)-7-MeC27, which had no significant effect on the bioactivity of 0-d-old female dummies as compared to the solvent treated controls (*p* = 0.08). The reason for this anomaly is unclear, particularly as both enantiomers of 7-MeC27 significantly disrupted responses from males when applied to male 0-d-old dummies. All compounds interrupting the pheromone response in *L. distinguendus* males when added to bioactive CHC profiles are minor components of the natural CHC profile of this species [31]. This suggests that it was not the appearance of a novel foreign compound but a shift in the ratios of familiar compounds what caused the loss of pheromone activity.

Disturbance of CHC profiles by the addition of synthetic compounds also has been demonstrated in the context of nestmate recognition in social insects. Addition of specific alkanes to the CHC profile of individual workers increased aggressive behavior by nestmates in some species (reviewed by van Zweden and d’Ettore [33]). Some studies provided evidence that methyl-branched alkanes might be more important in this respect than straight chain alkanes [10,12,13,21,55]. The results of the present study show that the addition of both straight chain and methyl-branched alkanes can disturb bioactive CHC profiles in the context of sexual communication (Figure 4a). In the context of nestmate recognition, it should be much easier to render a nestmate unacceptable by experimental manipulation of its CHC profile than the reverse, *i.e.*, rendering a non-nestmate acceptable. That is, if a nestmate recognizes a non-nestmate as foreign by perceiving the species-specific CHCs in ratios differing from the known colony blend, only the exact correction of the imbalance should render it acceptable. In contrast, many different compounds added to the cuticle of a nestmate could make it unacceptable. Transferring these considerations to the present study might explain why *L. distinguendus* responded only to 3-MeC27 in the pheromone restoration experiment (Experiment 1), whereas in the pheromone interruption experiment (Experiment 2) many different compounds disrupted the pheromonal response equally well. 

The striking parallels between the role of CHCs in nestmate recognition of social insects and sexual communication in *L. distinguendus* suggest that there might also be analogies in the sensory organs used to detect these compounds. It has been suggested that single CHCs used as sex pheromone components are perceived by gustatory sensilla. However, conventional gustatory sensilla have been predicted to be unsuitable for the perception of complex CHC profiles because they typically are innervated by only a small number of receptor neurons [15]. This idea was corroborated by the identification of a specialized olfactory sensillum type in the ant *Camponutus japonicus* which is innervated by about 130 olfactory receptor neurons and capable of discriminating complex CHC profiles originating from nestmates and non-nestmates, respectively [14,15]. Given the results of the present study suggesting that *L. distinguendus* wasps, like the ants, perceive CHC profiles as a whole, it will be interesting to determine whether similar specialized sensilla also are present on the antennae of this species. 

Apart from 7-MeC27 tested on 0-d-old female dummies, *L. distinguendus* males did not discriminate between the enantiomers of synthetic methylalkanes. However, in a previous study [3], males preferred (*S*)-3-MeC27 over (*R*)-3-MeC27 when presented in a different context, *i.e.*, when applied to filter paper together with a chemical background of the other CHCs and triacylglycerides. This preference was not seen in the present study when the enantiomers were tested with three-dimensional 4-d-old male dummies [3]. These results suggest that chemically-based sex recognition in *L. distinguendus* is supported by visual and/or tactile stimuli, as previously shown for the pteromalid wasps *Nasonia vitripennis* [56] and *Dibrachys cavus* [57]. Thus, the absolute configuration of 3-MeC27 occurring on the cuticle of *L. distinguendus* wasps could not be concluded unambiguously from the behavior of males as has been done, for instance, for the enantiomers of 5-MeC27 with the egg parasitoid *Oenocyrtus kuvanae* (Encyrtidae) [25]. Thus, the chirality of 3-MeC27 in *L. distinguendus* remains to be established by analytical methods. However, before this can be accomplished, methods need to be developed for resolving the enantiomers of methylalkanes or of determining their absolute configuration on microgram to nanogram scale [58,59].

## 5. Conclusions

Subsets of CHCs have evolved to have secondary functions as contact sex pheromones in several insect taxa [1]. However, details of the structure-activity relationships of CHCs and the contribution of individual components to the bioactivity of complex CHC profiles have been elucidated in very few species. It appears that the mechanisms for the perception of CHCs in the context of sexual communication differ significantly among taxa. In Coleoptera and Diptera, for instance, individual key components may elicit behavioral responses when presented alone [17,18,19,20,22,51,52]. In contrast, for *L. distinguendus*, CHC profiles are perceived as a whole and the key compound 3-MeC27 needs to be present with a chemical background of other cuticular lipids to elicit behavioral responses in males. Furthermore, the response elicited by an attractive individual could be disturbed by distortion of the CHC profile by enhancement of individual compounds. Given that purified CHC fractions also have been shown to elicit behavioral responses in other parasitic wasp species [60], further studies are needed to understand the role of individual CHCs in these species, and to investigate whether the perception mechanism found in *L. distinguendus* is common in parasitic wasps. 
